# Is perinatal asphyxia predictable?

**DOI:** 10.1186/s12884-020-02876-1

**Published:** 2020-03-30

**Authors:** Anna Locatelli, Laura Lambicchi, Maddalena Incerti, Francesca Bonati, Massimo Ferdico, Silvia Malguzzi, Ferruccio Torcasio, Patrizia Calzi, Tiziana Varisco, Giuseppe Paterlini

**Affiliations:** 1 Department of Obstetrics and Gynecology, ASST Vimercate, Carate B.za Hospital, University of Milano-Bicocca, Monza, Italy; 2 Department of Obstetrics and Gynecology, Fondazione MBBM, San Gerardo Hospital, University of Milano-Bicocca, Monza, Italy; 3 Department of Obstetrics and Gynecology, ASST Vimercate, Vimercate Hospital, Vimercate, Italy; 4 Neonatal Intensive Care Unit, Fondazione MBBM, San Gerardo Hospital, Monza, Italy; 5 Department of Pediatrics, ASST Vimercate, Carate B.za Hospital, Vimercate, Italy; 6 Department of Pediatrics, ASST Vimercate, Vimercate Hospital, Vimercate, Italy; 7 Department of Pediatrics, ASST Monza, Desio Hospital, Desio, Italy

**Keywords:** Hypoxic-ischemic encephalopathy; asphyxia; sentinel events, Nulliparity, Umbilical artery pH, Fetal heart rate monitoring

## Abstract

**Background:**

The objective of our study was to evaluate the association between perinatal asphyxia and hypoxic-ischemic encephalopathy (HIE) with the presence of ante and intrapartum risk factors and/or abnormal fetal heart rate (FHR) findings, in order to improve maternal and neonatal management.

**Methods:**

We did a prospective observational cohort study from a network of four hospitals (one Hub center with neonatal intensive care unit and three level I Spoke centers) between 2014 and 2016. Neonates of gestational age ≥ 35 weeks, birthweight ≥1800 g, without lethal malformations were included if diagnosed with perinatal asphyxia, defined as pH ≤7.0 or Base Excess (BE) ≤ − 12 mMol/L in Umbical Artery (UA) or within 1 h, 10 min Apgar < 5, or need for resuscitation > 10 min.

FHR monitoring was classified in three categories according to the American College of Obstetricians and Gynecologists (ACOG). Pregnancies were divided into four classes: 1) low risk; 2) antepartum risk; 3) intrapartum risk; 4) and both ante and intrapartum risk.

In the first six hours of life asphyxiated neonates were evaluated using the Thomson score (TS): if TS ≥ 5 neonates were transferred to Hub for further assessment; if TS ≥ 7 hypothermia was indicated.

**Results:**

Perinatal asphyxia occurred in 21.5‰ cases (321/14,896) and HIE in 1.1‰ (16/14,896). The total study population was composed of 281 asphyxiated neonates: 68/5152 (1.3%) born at Hub and 213/9744 (2.2%) at Spokes (*p* < 0.001, OR 0.59, 95% CI 0.45–0.79). 32/213 (15%) neonates were transferred from Spokes to Hub. Overall, 12/281 were treated with hypothermia. HIE occurred in 16/281 (5.7%) neonates: four grade I, eight grade II and four grade III. Incidence of HIE was not different between Hub and Spokes.

Pregnancies resulting in asphyxiated neonates were classified as class 1) 1.1%, 2) 52.3%, 3) 3.2%, and 4) 43.4%. Sentinel events occurred in 23.5% of the cases and FHR was category II or III in 50.5% of the cases. 40.2% cases of asphyxia and 18.8% cases of HIE were not preceded by sentinel events or abnormal FHR.

**Conclusions:**

We identified at least one risk factor associated with all cases of HIE and with most cases of perinatal asphyxia. In absence of risk factors, the probability of developing perinatal asphyxia resulted extremely low. FHR monitoring alone is not a reliable tool for detecting the probability of eventual asphyxia.

## Background

Cases of neonatal encephalopathy related to perinatal asphyxia are called hypoxic-ischemic encephalopathy (HIE) and can lead to long-term neurological sequelae and cerebral palsy [1, 2].

The Sarnat and Sarnat classification [3] is still the universally accepted scoring system to provide information about the prognosis for the asphyxiated neonate. This staging is based on the infant’s clinical presentation, examination findings and the presence of seizures, with emphasis on the duration of symptoms.

Both clinical (Thompson scale [4]) and instrumental (cerebral function monitor/aEEG) assessment are validated by international literature for classifying the severity of damage immediately after birth. This allows for initiating the correct treatment (ie Therapeutic Hypothermia) as soon as possible.

Perinatal asphyxia is defined as at least one of the following characteristics in a neonate: 10 min Apgar score ≤ 5, need for resuscitation > 10 min, metabolic acidosis (pH ≤7.0 or BE ≤-12 mMol/L in umbilical artery (UA) or within 1 h of life).

There is a dose-dependent relationship between the degree of acidosis and the likelihood of adverse neonatal outcome [5, 6].

Both ante and intrapartum risk factors have been associated with perinatal asphyxia and HIE. Some of these are clinically recognized as asphyxial birth events (sentinel events): uterine rupture, placental abruptio, shoulder dystocia and cord prolapse. However, the majority of risk factors (preeclampsia, intrauterine growth restriction, intrapartum fever, emergency delivery) seem to interact in the causal pathway to HIE rather than being its direct antecedent [7–11]. In addition to the historically described risk factors, new ones have recently been identified, such as obesity and previous cesarean section [12]. Nevertheless, results from a large registry of neonatal encephalopathy show that half of the neonates had neither asphyxia nor inflammatory indicators, suggesting that a large number of cases remain unexplained [13].

Fetal heart rate (FHR) monitoring provides important information about fetal status. However, its role in the prediction of fetal asphyxia remains controversial, despite efforts directed at its interpretation and the development of protocols for the management of abnormal patterns [14, 15].

To summarize, there are a number of conditions that have been associated with the presence of perinatal asphyxia and its extreme consequence, HIE. Nevertheless, it is problematic to identify the sequence of events that leads to perinatal asphyxia during prenatal and intrapartum care.

In this context the objective of our study was to evaluate the association between perinatal asphyxia and HIE with the presence of risk factors and/or abnormal FHR findings in order to improve maternal and neonatal management.

## Methods

We conducted a prospective observational cohort study between July 2014 and June 2016 from a network of four hospitals with different levels of care which shared protocols of intrapartum and neonatal care.

Inclusion criteria were asphyxiated neonates of gestational age ≥ 35 weeks, birthweight ≥1800 g, and no severe malformations. Perinatal asphyxia was defined as pH ≤7.0 or BE ≤-12 mMol/L in UA or within 1 h, 10 min Apgar ≤5, or need for resuscitation > 10 min [16].

The network includes one Hub University Hospital center with a neonatal intensive care unit that offers therapeutic hypothermia, and three level I Community Hospital Spoke centers.

In Spoke centers, only women with low risk pregnancies or minor complications can be admitted for delivery. High risk pregnancies for medical or fetal complications are referred to Hub. Patients with low risk pregnancies may choose to deliver in either Spoke or Hub centers.

Perinatal mortality rate in our network is superimposable to the general Italian one, that is 4 ‰.

The Italian childbirth system offers universal and free of charge maternity care.

The acid-base status is routinely determined at birth by UA and, if not feasible, within one hour of life on the neonate.

Data regarding maternal history, pregnancy complications, labor and delivery were collected.

The uterine contraction changes during labor were monitored and prospectively registered. Tachysystole is defined as ≥5 contractions in 10 min for 30 min or more.

Fetal electronic monitoring on admission and/or during labor was prospectively collected and managed using the Clark algorithm [17]. The FHR was monitored intermittently during the first and second stages of labor, at least every 15 min for 1 min in the active phase of the first stage of labor and after every contraction in the second stage. Continuous electronic monitoring was implemented in the presence of obstetric or medical risk factors evaluated on admission and during labor, or if the FHR was abnormal at intermittent auscultation. The last hour tracing was reviewed by two authors (FB, MI), blinded to the outcome of the neonate, and it was classified in 3 categories according to ACOG classification. Under this classification system, category I tracings were defined as including all of the following characteristics: baseline rate between 110 and 160 beats per minute, moderate variability, and absence of late or variable decelerations. Category III tracings were defined as absent variability with recurrent late decelerations, recurrent variable decelerations, or bradycardia or sinusoidal pattern. Category II tracings include those patterns that did not meet the criteria of categories I or III. Discrepancies in category status of tracing were adjudicated by an additional reviewer (AL) blinded to the assessments of the initial reviewers.

We divided pregnancies into four classes: low risk (no risk factors); antepartum risk (nulliparous women, maternal age > 40 years, BMI > 30, oligohydramnios or polyhydramnios, fetal growth restriction, induction of labor, maternal disease, previous cesarean); intrapartum risk (sentinel event, thick meconium during labor, fever, tachysystole); and both ante and intrapartum risk.

Data recorded on each infant included: pH, BE, lactate, need for ventilation by mask or endotracheal tube during initial stabilization, need for cardiac massage, biochemical and instrumental data. In the first six hours of life, asphyxiated neonates were monitored with the Thomson score [4]: if TS was ≥5, the neonate was transferred to the Hub for further surveillance; if TS was ≥7 hypothermia was indicated.

Encephalopathy was classified according to Sarnat & Sarnat [3].

Neurodevelopmental follow-up was offered to all neonates until 1 year of age and to neonates with moderate or severe encephalopathy until 6 years of age.

Written informed consent was obtained from all participants.

Descriptive statistics were calculated as mean (standard deviation) for numerical variables and absolute frequencies and percentages for categorical variables. Statistical analysis included univariate analyses with Student T-test, one-way ANOVA, chi-square and Fisher exact test. Multivariate logistic regression analyses were performed to control for confounding variables (SPSS). A two-tailed *p* value <.05 was considered significant. SPSS (IBM Corp. version 24) was used to run the analyses.

## Results

During the study period perinatal asphyxia occurred in 321/14,896 cases (21.5‰) and HIE in 16/14,896 (1.1 ‰).

Asphyxia occurred in 1.5% (79/5152) in the Hub center, in 1.8% (64/3552) in Spoke 1, 2.9% (94/3269) in Spoke 2 and 2.9% in Spoke 3 (84/2923).

We excluded 40 neonates because they did not meet the inclusion criteria (two cases of neonatal diagnosis of metabolic disease), or because we did not have parental consent, or we had incomplete data for enrolment. Thus, 281 asphyxiated neonates were included in the analysis: 68/5152 (1.3%) delivered at the Hub and 213/9744 (2.2%) in Spoke centers (*p* < 0.001, OR 0.59, 95% CI 0.45–0.79).

Of the 281 asphyxiated neonates, 80/281 had UA pH ≤ 7 (28%), 261/281 had BE ≤ − 12 (93%) (of which 51 (18%) with BE ≤ − 16); 189/281 (67%) neonates were enrolled only for BE ≤ − 12.

There were no cases of HIE among neonates who were not enrolled.

There were no perinatal deaths during the study period.

Descriptive characteristics of asphyxiated neonates are reported in Table 1.

**Table 1:** Neonatal characteristics

	N (%)	Mean ± SD (range)
Male	154 (55)	
Birth weight (g)		3261 ± 435 (4400–2020)
pH UA		7.05 ± 0.11 (7.34–6.66)
pH UA ≤ 7	80 (28.5)	
BE UA		−14.1 ± 2.3 (− 5.3 - -22)
BE UA ≤ − 12	261 (92.8)	
BE UA ≤ − 16	51 (18.1)	
Lactate UA (mmol/L)		10.5 ± 2.8 (18.4–3.7)
5 min Apgar < 5	16 (5.7)	
10 min Apgar < 5	5 (1.8%)	
Neonatal resuscitation > 10’	17 (6)	

Legend: *UA* Umbilical Artery, *BE* Base Excess

During neonatal monitoring TS was ≥5 in 24/281 cases (8.5%) and 32/213 (15%) neonates were transferred from Spokes to the Hub Center.

**Fig. 1:**
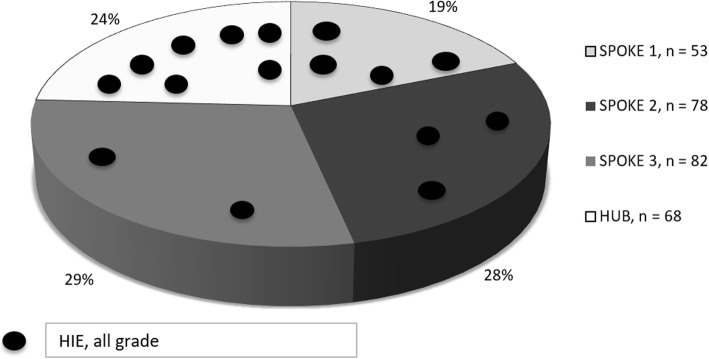
Asphyxia and hypoxic-ischemic encephalopathy according to place of birth

Overall, 12/281 neonates received hypothermia. There were 9/281 cases of neonatal seizures. HIE was diagnosed in 16/281 (5.7%,) including four with grade I, eight with grade II, and four with grade III HIE. Asphyxia and HIE according to place of birth are shown in Fig. 1. No differences in incidence of HIE were observed between Hub and Spokes: 7/5152 (0.13%) vs 9/9744 (0.09%), *p* = 0.58, OR = 1.4 95% CI 0.44–49. The detail of every case is reported in Table 2.

**Table 2:** Description of cases of HIE

Case	Parity	Antepartum Risk	GA (wks)	Intrapartum Risk	Mode of Delivery	FHR cat II-III ACOG	Neonatal weight (gr)	UA pH	UA BE	10 min Apgar	Neonatal Resuscitation	Hypothermia	HIE (grade)	Neonatal outcome
1	0	Obesity Nulliparity	39, 3	Tachysystole	Kristeller maneuver	Yes	2910	6.96	−17.7	10	No	No	I	Normal
2	0	Nulliparity	35, 6	–	Kristeller maneuver	No	2360	7.09	−13	9	No	No	I	Normal
3	0	3 previous CS Nulliparity	37	Placental Abruption	Urgent CS befour labour	No	3150	6.8	−22	5	Yes	No	I	Normal
4	0	Nulliparity	40, 2	–	CS in labour	Yes	2490	6.97	−9	8	No	No	I	Normal
5	0	Nulliparity Preeclampsia Induction of labour	39, 4	–	OVD	Yes	3430	6.85	−22	6	Yes	Yes	II	Normal
6	2	Age 42 yrs. Obesity Gestational diabetes Polyhydramnios	39, 1	Bradycardia	Kristeller maneuver	Yes	3440	6.8	−13.4	7	Yes	Yes	II	Normal
7	0	1 previous CS Nulliparity	38, 5	–	CS in labour	No	3150	6.86	−16.3	6	Yes	Yes	II	Normal
8	0	Nulliparity	40	–	Spontaneous VD	Yes	2900	7.02	−16.4	4	Yes	Yes	II	Normal
9	1	Hypothyroidism	39, 4	Shoulder dystocia	Spontaneous VD	No	3800	6.97	−12.1	4	Yes	Yes	II	Normal
0	1	Complete placenta praevia	37, 4	Intrapartum hemorrhage	Elective CS	No	3500	7.23	−5.3	5	Yes	Yes	II	Normal
11	0	Nulliparity	39, 6	Tachysystole	Spontaneous VD	No	3250	7.15	−10.5	8	Yes	Yes	II	Normal
12	1	Hypothyroidism Nulliparity	39, 6	Bradycardia	CS after failure of OVD	Yes	3690	6.83	−22	7	Yes	Yes	II	Normal
13	0	Obesity Nulliparity Oligohydramnios Induction of labour	41, 3	Tachysystole	OVD	Yes	3140	6.8	nv	8	No	Yes	III	Cerebral Palsy
14	1	1 previous CS Polyhydramnios	40, 1	Tachysystole	OVD	Yes	3400	7	−13	7	Yes	Yes	III	Cerebral Palsy
15	0	1 previous CS Nulliparity Oligohydramnios Induction of labour	40	Uterine rupture	Urgent CS befour labour	Yes	3070	6.66	−22	1	Yes	Yes	III	Cerebral Palsy
16	0	Nulliparity Polyhydramnios	40, 3	Bradycardia	Urgent CS befour labour	Yes	2990	6.8	nv	4	Yes	Yes	III	Cerebral Palsy

Legend: *CS* Caesarean Section, *GA* Gestational Age, *VD* Vaginal Delivery, *OVD* Operative Vaginal Delivery, *FHR* Fetal Heart Rate, *ACOG* American College of Obstetricians and Gynecologists, *UA* Umbilical Artery, *BE* Base Excess, *HIE* Hypoxic-Ischemic Encephalopathy

**Fig. 2:**
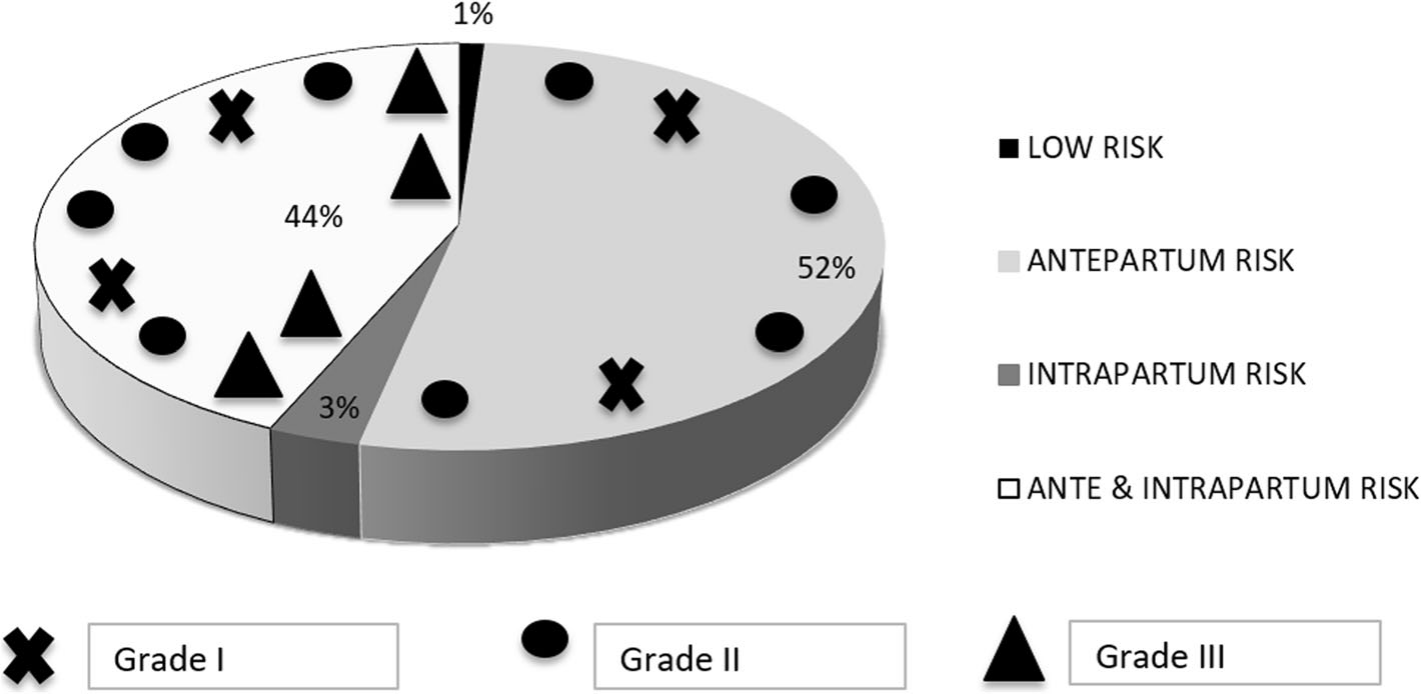
Cases of hypoxic-ischemic encephalopathy and grade in asphyxiated neonates according to presence of risk factors

Pregnancies were classified as low risk in 3/281 (1.1%) cases, with antepartum risk in 147/281 (52.3%), intrapartum risk in 9/281 (3.2%), and both ante and intrapartum risk in 122/281 (43.4%) cases (Fig. 2).

There were 214/281 (76.2%) nulliparous women equally distributed in class 2 and 4, 16/281 (5.7%) women > 40 years, 87/281 (31.0%) with BMI > 30, 14/281 (5.0%) cases of oligohydramnios, 20/281 (7.1%) cases of polyhydramnios and 8/281 (2.8%) cases of fetal growth restriction. We recorded 18/281 (6.4%) cases of hypertensive disorders of pregnancy, 4/281 (1.4%) positive thrombophilic status, 5/281 (1.8%) pre-gestational and 31/281 (11.0%) gestational diabetes mellitus, 25/281 (8.9%) thyroid diseases, 8/281 (2.8%) gestational cholestasis, 1/281 (0.4%) antepartum hemorrhage and 19/281 (6.8%) previous cesarean section.

Labor was induced in 96/281 (34.2%) of cases. Thick meconium during labor was observed in 21/281 cases (7.5%), fever or chorioamnionitis in 17/281 (6.0%) and tachysystole in 52/281 (18.5%).

**Fig. 3:**
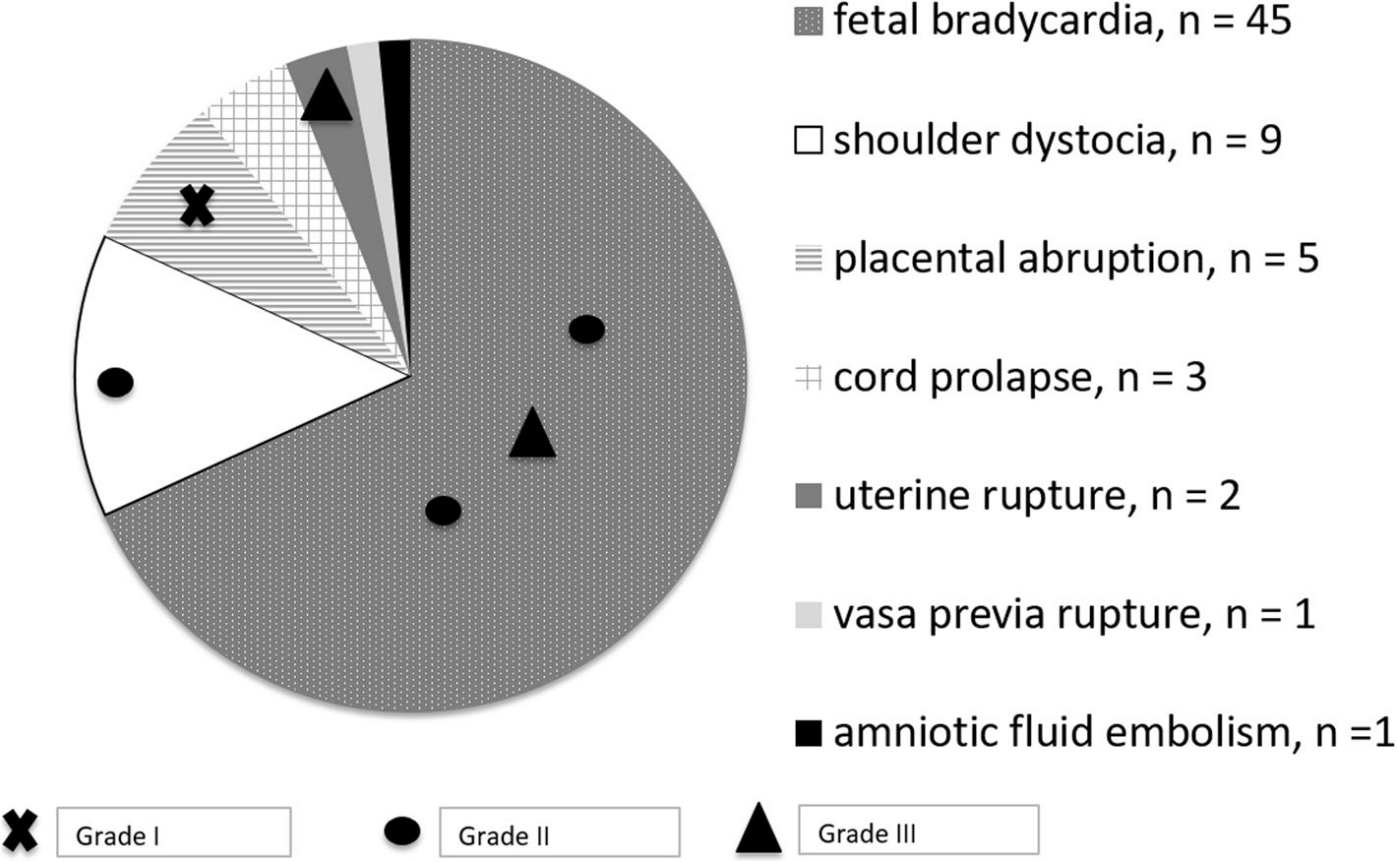
Cases of hypoxic-ischemic encephalopathy and grade in asphyxiated neonates according to presence of sentinel events

Sentinel events occurred in 66/281 cases (23.5%) and preceded HIE in 6/16 (37.5%), as described in detail in Fig. 3. Incidence of sentinel events was not different among neonates with HIE and others (*p* = 0.4089).

Only three cases of asphyxia (1%) and no cases of HIE occurred in class 1 (low risk pregnancies). There were no cases of UA pH ≤ 7 in this class.

There were 67/281 (24%) operative vaginal deliveries and 56/281 (20%) cesarean sections including 39/56 (70%) because of fetal distress. FHR category II and III in the last hour before delivery were documented in 142/281 (50.5%) cases of perinatal asphyxia, and they preceded HIE in 9/16 (56.3%) respectively. In 7/142 cases, FHR category II or III was observed before labor.

Moreover, FHR category II or III occurred in 61/80 (76%) cases with UA pH ≤ 7 compared with 81/201 (40%) cases with UA pH > 7 (*p* < 0.001 OR 4.8 95% CI 2.6–8.9). HIE occurred more often if UA pH was ≤7 (12/80, 15.0%) in respect to case with UA pH > 7 (4/137, 3.0%) (*p* = 0.002, OR 5.9 95% CI 1.7–22.5).

113/281 (40.2%) cases of asphyxia and 3/16 (18.8%) cases of HIE were not preceded by sentinel events or abnormal FHR findings.

## Discussion

In our network the rates of neonatal asphyxia (1%), as well as of pH ≤ 7 (5‰) and HIE (0.7–1.7‰) were comparable to those described in literature when similar definitions are used [5, 6, 18–22].

As expected, only a minority of asphyxiated neonates developed HIE (5.7%) and they were promptly identified. Thanks to our shared care protocols and cooperative management system, appropriate care was guaranteed for all asphyxiated neonates, while minimizing the number of newborns transferred.

In our series the number of sentinel events in cases of perinatal asphyxia and HIE was as high as previously described in literature (15–35%) [7, 13]. Emergency delivery can change the prognosis of these events with an appropriate diagnosis, management and team organization. Nevertheless, in some cases the clinical presentation reflects a chronic damage that has already occurred instead of an acute event (i.e. placental abruption), limiting the therapeutic options. In other cases, when a non-reassuring signal occurs (i.e. bradycardia), the available time to prevent fetal damage may be too short to alter the outcome (i.e. uterine rupture).

Many authors have focused on searching for risk factors associated with perinatal asphyxia and neonatal encephalopathy: a wide number of antecedents has been identified, nonetheless a variable percentage of the events remain unexplained [8, 9, 11]. In order to classify pregnancies into different classes of risk, we prospectively collected all known risk factors in our population, including the new emerging ones (eg. previous cesarean section, obesity). Low risk pregnancies were only a minor proportion (1.1%) of our population and no cases of HIE presented in this group. This means that we managed to identify at least one risk factor (ante and/or intrapartum) associated with every case of HIE and of perinatal asphyxia, except 3 cases of perinatal asphyxia that did not have any identified risk factor. Therefore, we can assume that at least one of the risk factors we examined underlies the phenomenon of perinatal asphyxia even if we cannot always identify a causal relation. More importantly, the probability of developing perinatal asphyxia in this low-risk population appears to be extremely low.

Our study design does not allow us to assign a relative risk or hazard ratio to the risk factors recorded.

The most common risk factor for asphyxia is nulliparity. It is identified in 76% of cases (51% in our general population) and it is the only risk factor in 16% of cases. This is obviously a non-modifiable condition and it is widespread in the general obstetric population. Nonetheless, it has been previously described in association with need for cooling [21, 22]. The role of nulliparity could be explained by the increased frequency of pregnancy complications related to it, such as defective placentation and impaired fetal growth at term, undetected by routine clinical exams. Moreover, because of the longer duration of labor, nulliparas are at greater risk of infection and fetal intolerance to labor, interventions, operative deliveries, and associated perinatal events.

In spite of this, a recent study of Liljestrom [23] reported that in HIE cases an obstetric emergency occurred more commonly in the parous than in the nulliparous women, suggesting that intrapartum complications can occur also in women at lower risk, representing a major challenge in obstetrics.

The second most frequent risk factor is fetal tachysystole, which presented in 52/281 cases (18.5%). In most cases, tachysystole was associated with other risk factors, such as the need for induction of labor (23 cases) and/or oxytocin augmentation (29 cases). Use of oxytocin can lead to excessive uterine activity, which has been associated with neonatal morbidity both in spontaneous and induced labor [24]. Unlike nulliparity, this is a modifiable risk factor that can be prevented by using different labor management. The use of a check-list to prevent and treat tachysystole, as suggested by some authors [25], could be a helpful tool. Interestingly, in our series, tachysystole was not linked to FHR ACOG Category II-III in 22 cases (42%), but it still resulted in asphyxia.

From the review of FHR tracing prospectively collected and managed according to ACOG algorithms, we observed that FHR Category II or III occurred in 50.5% of asphyxiated neonates. It preceded HIE in 56.3% of cases.

As recently reported [14], we acknowledge the limitations of electronic FHR monitoring in predicting perinatal asphyxia and the poor specificity of this test. Interestingly, our findings are similar to those of Clark, who using the same classification, observed that of infants who are born with metabolic acidemia, only approximately one-half could be identified with FHR monitoring.

Independently from the efforts at training and at applying uniform algorithms during labor, it may be unrealistic to expect high sensitivities for the detection of asphyxia using FHR alone. This underscores thefact that key management decisions in preventing asphyxia should not be based only on FHR interpretation. Other factors, including maternal and fetal history, stage and progress of labor and signs of fetal distress in labor, should be taken into account. Some Authors have suggested that a physiological interpretation of the FHR recordings may improve our understanding offetal oxygenation during labor [26, 27]. Whether such interpretation may prevent asphyxia is unproven.

Our study has some limitations. First of all, the number of cases included in the study is not large. In addition, some cases were excluded due to lack of information or refusal of consent. The strengths of our study are that umbilical cord gas analysis was done consistently across the participating institutions; and the prospective nature of the study, with hospitals using shared protocols of maternal and neonatal care, as well as of methods of induction, labor management, oxytocin use, and FHR tracing interpretation.

Furthermore, all FHR tracings were reviewed in order to standardize their interpretations. In addition, for every neonate we have the values of both pH and BE and lactate, thus permitting to have a more realistic picture of all nuances of acid-base status, and not only the most extreme. In our network, all cases of HIE were promptly identified with a limited number of newborns transferred. Shared care protocols and cooperative management system has guaranteed appropriate care for all asphyxiated neonates. We hope that long-term neurodevelopmental follow up data will help us to better define risk factors for neurological sequelae in asphyxiated neonates, allowing the development of adequate rehabilitation programs for these children.

## Conclusions

We identified at least one risk factor associated with all cases of HIE and with the majority of cases of perinatal asphyxia. In the absence of risk factors, the probability of developing perinatal asphyxia resulted extremely low. Among risk factors, the most frequent were nulliparity and tachysystole. Sentinel events confirmed their significant role as antecedents of asphyxia. Our findings on FHR tracing support that it is unrealistic to expect high sensitivities for the detection of asphyxia using FHR alone. FHR interpretation should consider the background risk factors. Future studies will address the question of whether a more integrated approach taking into account risk factors and new adjunctive monitoring methods can improve awareness of fetal status and, consequently, prediction of asphyxia.

## Data Availability

Data on individual patient are available upon request.

## Abbreviations

HIE: Hypoxic-ischemic encephalopathy
FHR: Fetal heart rate
BE: Base Excess
UA: Umbilical Artery
ACOG: American College of Obstetricians and Gynecologists
TS: Thomson score
CS: Caesarean Section
GA: Gestational Age
VD: Vaginal Delivery
OVD: Operative Vaginal Delivery
